# Deficiencies in Natural Killer Cell Numbers, Expansion, and Function at the Pre-Neoplastic Stage of Pancreatic Cancer by KRAS Mutation in the Pancreas of Obese Mice

**DOI:** 10.3389/fimmu.2018.01229

**Published:** 2018-06-21

**Authors:** Kawaljit Kaur, Hui-Hua Chang, Paytsar Topchyan, Jessica Morgan Cook, Andre Barkhordarian, Guido Eibl, Anahid Jewett

**Affiliations:** ^1^Division of Oral Biology and Oral Medicine, School of Dentistry and Medicine, University of California, Los Angeles, Los Angeles, CA, United States; ^2^Department of Surgery, David Geffen School of Medicine, University of California, Los Angeles, Los Angeles, CA, United States; ^3^Department of Tumor Immunology, School of Dentistry and Medicine, University of California, Los Angeles, Los Angeles, CA, United States; ^4^UCLA Jonsson Comprehensive Cancer Center, Los Angeles, CA, United States

**Keywords:** natural killer cells, KRAS, IFN-γ, HFCD, control diet, pre-neoplastic lesions

## Abstract

The combined/synergistic effect of genetic mutation of KRAS in the pancreas and obesity, a life-style factor on suppression of natural killer (NK) cells at the pre-neoplastic stage of pancreatic cancer has not been investigated and is the subject of this report. Obese mice with KRAS (KC) mutation in the pancreas fed with high-fat calorie diet (HFCD) exhibit severe deficiencies in the NK cell expansion and function at the pre-neoplastic stage of pancreatic cancer. Decreased NK cell-mediated cytotoxicity is observed in the peripheral blood, spleen, pancreas, and peri-pancreatic adipose tissue in obese KC mice, whereas in bone marrow an increased NK cell-mediated cytotoxicity is observed when compared to lean WT mice fed with control diet (CD). Obese KC mice on HFCD demonstrated the least ability to expand NK cells or induce NK cell-mediated cytotoxicity when compared to the other groups of mice. Indeed, the following profile WT/CD > WT/HFCD > KC/CD > KC/HFCD was seen for the ability to expand NK cells or mediate cytotoxicity among four groups of mice in spleen, peripheral blood, pancreas, and peri-pancreatic adipose tissue. Sorted NK cells from the splenocytes of four groups of mice also exhibited the same profiles for the cytotoxicity as the unsorted splenocytes, and a decreased IFN-γ secretion could be seen in cultures of NK cells from KC mice fed with either CD or HFCD. Cultures of NK cells with autologous monocytes from obese KC mice fed with HFCD exhibited decreased cytotoxicity and IFN-γ secretion, whereas cultures of allogeneic NK cells from WT mice fed with CD with osteoclasts of obese mice fed with HFCD demonstrated decreased cytotoxicity but augmented IFN-γ secretion. Increased IL-6 along with decreased IFN-γ and cell-mediated cytotoxicity by the NK cells, within NK-adipose tissue of KC/HFCD mice, may provide safe microenvironment for the expansion of pancreatic tumors.

## Highlights

- KRAS mutation of pancreas and/or feeding with HFCD is associated with the significant loss of NK cell numbers and function
- NK cell defect in mice with KRAS mutation and/or fed with HFCD occurs at the pre-malignant stage of pancreatic cancer
- NK cell defects are seen during activation of NK cells by autologous monocytes in mice with KRAS mutation and/or fed with HFCD
- Osteoclasts from mice with KRAS mutation and/or fed with HFCD have decreased RAE1-delta activating as well as inhibitory MHC-class I surface receptors, and are less able to expand NK cells
- Decreased killing but augmented IFN-γ is observed when allogeneic NK cells obtained from WT mice interact with osteoclasts from KC mice on HFCD.

## Summary

We have previously shown that NK cells select and differentiate cancer stem cells (CSCs)/undifferentiated tumors by direct killing of CSCs and secretion of IFN-γ which lead to differentiation of CSCs, respectively. These are two important functions of NK cells which prevent establishment and progression of cancer. Here, we show defects in expansion, killing, and secretion of IFN-γ by NK cells in mice which have KRAS mutation in pancreas and/or are fed with HFCD at the pre-cancerous stage. NK cell-mediated killing is always defective in these mice; however, depending on the extent of defect in either NK cells or in the cells that interact with NK cells or both, the secretion of IFN-γ could be variable effecting differentiation of cancer stem cells. Increased IL-6 secretion in the presence of decreased IFN-γ secretion induced by surrounding or infiltrating adipose tissue within tumor microenvironment may constitute a significant driving force in the expansion of pancreatic tumors.

## Introduction

Pancreatic ductal adenocarcinoma (PDAC) constitutes over 90% of pancreatic cancers, and is the fourth most severe cause of cancer-related deaths worldwide, with a 5-year survival rate of less than 5% (1–5). The genes mutated in pancreatic cancer include KRAS2, p16/CDKN2A, TP53, and SMAD4/DPC4 in combination with genomic and transcriptomic alterations facilitate cell cycle deregulation, cell survival, invasion, and metastases (2, 6, 7). In most cases, PDAC is initiated by the oncogenic mutant KRAS which has been shown to drive pancreatic neoplasia (8). KRAS mutations are associated with reduced patient survival in both malignant exocrine and PDAC (9, 10).

Obesity can lead to intra-pancreatic fatty infiltration and is associated with an increased risk of pancreatic cancer and its precursor lesions (11–13). Because of high prevalence of obesity in the United States, greater attention must be given to its prevention and management (14). Impaired immune function in the presence of excess adipose tissue has been demonstrated both in humans and genetically obese rodents (15–17). It was previously shown that high-fat diet significantly elevated the pancreatic neoplasm incidence (18, 19). However, it’s effect on natural killer (NK) cell expansion and function which constitutes first line of defense against establishment and progression of pancreatic tumors has not been clearly established.

Natural Killer cells are large granular lymphocytes functioning at the interface of innate and adaptive immunity (20, 21). NK cells develop in the bone marrow. The frequencies of NK cells within the lymphocyte populations are heterogeneous in different tissues, constituting about 3–10% of circulating lymphocytes and those within the secondary lymphoid organs (22, 23). Early mature murine NK cells are identified by their surface expression of CD49b (DX5) or NK1.1 (24). The majority of DX5 expressing mature NK cells are found in various lymphoid and non-lymphoid peripheral tissues (25, 26). The activation of NK cells is controlled by the balance between the activating and inhibitory receptors (27–32). The activating receptors expressed by NK cells commonly recognize host or pathogen glycoproteins, and inhibitory receptors commonly recognize MHC-class I molecules (29, 33–35). NK cells recognize CSCs and virus-infected cells without prior sensitization both in humans and mice (36–39), targeting them by releasing pre-formed granules of proteins perforin and granzyme B, which can induce necrotic as well as apoptotic cell death in target cells (40–42). NK cells or their precursors move from the spleen toward pancreas, where they display an activated phenotype, exhibiting higher IFN-γ secretion and upregulation of CD69 and CD25 activation receptors (43). A reason for pancreatic cancer’s resilience toward intensive treatments is its ability to inhibit the immune system in such a way that during disease progression the immune system becomes permissive to tumor establishment, instead of eliminating it (44). It is suggested that fatty infiltration of pancreas is a risk factor for the induction and progression of PDAC (45, 46). In addition to secreting IL-6, peri-pancreatic and pancreatic adipose tissues contribute to the production of a number of inflammatory cytokines and growth factors which may drive the growth and expansion of pancreatic tumors (47–49). However, the underlying mechanisms of the contribution of fatty infiltrate in tumor induction have not been elucidated. High numbers of NK cells contribute to better prognosis in pancreatic adenocarcinoma patients (50).

We have recently shown that a decrease in cytotoxicity and lower IFN-γ secretion by osteoclast-expanded NK cells both from pancreatic cancer patients and tumor-bearing humanized BLT (hu-BLT) mice correlates with faster expansion of residual contaminating T cells within purified NK cells, whereas osteoclast-expanded NK cells from healthy human donors’ and hu-BLT mice with no tumors continue expanding super charged NK cells, while limiting T cell expansion for up to 30 to 60 days (51). We have also shown that expansion of T cells correlates with the decline in NK cell-mediated cytotoxicity and secretion of key cytokines, which could be one potential mechanism by which the numbers and function of NK cells decline in pancreatic cancer patients and in tumor-bearing hu-BLT mice (51). NK cell function is significantly downregulated following exposure to cancer cells in humans (31, 51–53) and mice (54, 55), as demonstrated by lower expression of activating surface receptors, decreased cytotoxic granule release and cytotoxicity, and reduced secretion of TNF-α and IFN-γ (56, 57).

In this paper, we demonstrate the association of both KRAS mutation in the pancreas and obesity with the increased loss of NK cell function at the pre-malignant stage of pancreatic tumorigenesis, likely paving the road for the establishment, invasion, and metastasis of pancreatic tumors. Potential contribution of IL-6 induced by peri-pancreatic adipose tissue in NK cell suppression is also shown and discussed.

## Materials and Methods

### Conditional KRAS(G12D) Mouse Model and Experimental Diet

To study the effect of obesity and high-fat calorie diet (HFCD) on NK function during pancreatic cancer development, the conditional KRAS(G12D) model was used (58). After weaning, offspring of *LSL-KRAS(G12D)* and *p48-Cre* (*or PDX-1-Cre*) mice were fed either a HFCD or a lean control diet (CD) for 3–4 months. The exact composition of the diets was previously described (59). The diets were obtained from Dyets, Inc., Pennsylvania. After 3–4 months, mice were euthanized, and the entire pancreas, peri-pancreatic adipose tissues, and other organs were harvested. Formalin-fixed, paraffin-embedded tissues were sectioned (4 µm) and stained with H&E. Sections of pancreatic tissues were histologically evaluated by a gastrointestinal pathologist for the presence and stage of murine PanIN lesions as described previously (55, 60). Animal studies were approved by the Chancellor’s Animal Research Committee (ARC) of the University of California, Los Angeles in accordance with the NIH Guide for the Care and Use of Laboratory Animals. Genotyping analysis was performed as described previously (61).

### Preparation of Single Cell Suspension

To prepare a single cell suspension of mice tissues for subsequent analyses, animals were sacrificed and spleen, pancreas, bone marrow from femur, peri-pancreatic fat and peripheral blood were harvested. Bone marrow cells and splenocytes were isolated, as described previously (62). The pancreas and adipose tissues were immediately cut into 1 mm^3^ pieces and placed into a digestion buffer containing 1 mg/ml collagenase IV, 10 U/ml DNAse, and 1% bovine serum albumin (BSA) in DMEM media and incubated for 20 min at 37°C oven on a 150-rpm shaker. After digestion, the sample was filtered through a 40 µm cell strainer and centrifuged at 1,500 rpm for 10 min at 4°C. The pellet was re-suspended in DMEM media and cells were counted. PBMCs were obtained using Ficoll-Hypaque centrifugation of heparinized peripheral blood specimens. The buffy coat containing PBMCs were harvested, washed, and re-suspended in RPMI 1640 medium.

### Cell Lines, Reagents, and Antibodies

RPMI 1640 supplemented with 10% fetal bovine serum (FBS) was used for culturing mouse NK cells, T cells, monocytes, BM cells, splenocytes, PBMCs, pancreas, and adipose cells. ST63, PanIN, and KC tumor cells were cultured in RPMI 1640 supplemented with 10% FBS. Lipopolysaccharides were purchased from Sigma-Aldrich (St. Louis, MO, USA). Recombinant IL-2 was obtained from NIH-BRB. Flow antibodies used in this study were purchased from Biolegend (San Diego, CA, USA). The mouse NK cells and monocyte purification kits were obtained from Stem Cell Technologies (Vancouver, BC, Canada).

### Cell Isolations and Cultures

Natural killer cells were isolated from splenocytes using mouse NK cells isolation kit (Stem-cells Technologies, Canada). Monocytes were isolated from BM cells using monocyte isolation kit (Stem-cells Technologies, Canada). The purity of NK cells and monocytes was greater than 90% based on flow cytometric analysis of DX5 and F4/80 antibodies, respectively.

### Bacteria Sonication

sAJ2 is a combination of eight different strains of sonicated Gram-positive probiotic bacteria (*Streptococcus thermophiles, Bifidobacterium longum, Bifidobacterium breve, Bifidobacterium infantis, Lactobacillus acidophilus, Lactobacillus plantarum, Lactobacillus casei, and Lactobacillus bulgaricus*) as demonstrated previously (63). For sonication, AJ2 was weighed and re-suspended in RPMI 1640 medium containing 10% FBS at a concentration of 10 mg/ml. The bacteria were thoroughly vortexed, then sonicated on ice for 15 s, at 6–8 amplitudes. Sonicated samples were then incubated for 30 s on ice. After every five pulses, a sample was taken to observe under the microscope until at least 80% of cell walls were lysed. It was determined that approximately 20 rounds of sonication/incubation on ice were conducted to achieve complete sonication. Finally, the sonicated samples (sAJ2) were aliquoted and stored in a −80°C freezer until use.

### Generation of Osteoclasts and Expansion of NK Cells

Purified mouse monocytes from BM cells were cultured in alpha-MEM medium supplemented with Macrophage Colony Stimulating Factor (M-CSF) (25 ng/ml) and Receptor Activator of Nuclear Factor Kappa-Β ligand (RANKL) (25 ng/ml). Medium was refreshed every 3 days with fresh alpha-MEM supplemented with M-CSF and RANKL. Purified NK cells from mice splenocytes were activated with IL-2 (10,000 U/ml) for 18–20 h before they were co-cultured with osteoclasts and sonicated AJ2 (NK:OCs:sAJ2 was 1:0.5:2) or LPS (100 ng/ml). The culture media was refreshed with RPMI 1640 medium supplemented with IL-2 every 3 days as described previously (51).

### Surface Staining

The cells were washed twice using ice-cold PBS with 1% BSA. Predetermined optimal concentrations of specific mouse monoclonal antibodies were added to 1 × 10^5^ cells/50 μl of cold 1% BSA + PBS and cells were incubated on ice for 30 min. Thereafter, cells were washed in cold 1% BSA + PBS and brought to 500 µl with 1%BSA + PBS. Staining was performed as described previously (64) and flow cytometric analysis was performed using Beckman Coulter Epics XL cytometer (Brea, CA) and results were analyzed in FlowJo vX software (Ashland, OR, USA).

### ELISA

Single ELISAs were performed and standard curve was generated as described previously (65). A standard curve was generated by either two- or threefold dilution of recombinant cytokines provided by the manufacturer to analyze and obtain the cytokine and chemokine concentration.

### Multiplex Cytokine/Chemokine Assay

The levels of cytokines and chemokines were examined by a commercially available multiplex assay (MILLIPLEX^®^ MAP Mouse Cytokine/Chemokine Magnetic Bead Panel, EMD Millipore, Billerica, MA, USA). A standard curve was generated by threefold dilution of recombinant cytokines provided by the manufacturer to determine the cytokine concentrations. The data were analyzed by the proprietary software (xPONENT 4.2, Millipore, Billerica, MA, USA).

### ^51^Cr Release Cytotoxicity Assay

The 4-h ^51^Cr release assay was performed, as demonstrated previously (66). Briefly, different numbers of effector cells were incubated with ^51^Cr-labeled ST63 target cells for 4 h. ST63 was previously used as specific targets of NK cells (55, 67). After that, the supernatants were harvested from each sample and released radioactivity was counted using the gamma counter. The percentage (%) specific cytotoxicity was calculated as follows:

$$\%\text{Cytotoxicity}=\frac{\text{Experimental cpm}-\text{spontaneous cpm}}{\text{Total cpm}-\text{spontaneous cpm}}$$

Lytic units (LU) 30/10^6^ was calculated by using the inverse of the number of effector cells needed to lyse 30% of tumor target ST63 cells × 100.

### Statistical Analysis

Prism 7 software was used for the statistical analysis. One-way ANOVA using Turkey’s multiple comparison tests was used to compare different groups. For cytotoxicity and cytokine analysis either duplicate or triplicate samples were used for each experiment. *n* denotes the number of mice used for the experiments. The following symbols represent the levels of statistical significance within each analysis, ***p-value <0.001, **p-value 0.001–0.01, *p-value 0.01–0.05.

## Results

### Decreased Percentages of DX5^+^ NK Cells and NK Cell Cytotoxic Function in KC Mice Fed With HFCD

We have recently demonstrated that KC mice fed with HFCD exhibited increased body weight as well as augmented visceral adipose tissue (68) and generated significantly more advanced pre-cancerous PanIN-2 and -3 lesions when compared to KC mice on CD (55). No invasive PDAC could be found in KC mice fed with either CD or HFCD at 3–4 months. No pancreatic neoplastic lesions were found in WT mice fed with either CD or HFCD. In addition, KC mice fed with HFCD had significantly more inflammation, acinar cell loss, and increased pancreatitis score as compared to KC mice fed with CD. The numbers of normal ducts within pancreas was much less in KC mice fed with HFCD when compared to those fed with CD, and pancreatic fibrosis was only observed in KC mice and not in WT mice (55).

To evaluate the effect of KRAS mutation and HFCD, we determined the total numbers of CD45+ immune cells, percentage of DX5+ NK cells, and total numbers of NK cells from different tissue compartments of WT and KC mice (Figure S1 in Supplementary Material). On average, no statistically significant differences could be observed in the number of cultured CD45+ immune cells from different tissues between the four groups of mice (Figures S1A,B in Supplementary Material). When the percentages of DX5+ NK cells were determined in the spleen, PBMCs, pancreas, and adipose tissues after culture, there was a consistent and significant decline in the percentages of DX5+ NK cells within WT mice fed with HFCD or KC mice fed with CD as well as HFCD, exhibiting the following profiles (WT/CD > WT/HFCD > KC/CD > KC/HFCD) (Figure S1A in Supplementary Material). The most severe decline was seen in KC mice fed with HFCD (Figure S1A in Supplementary Material). Statistically significant differences in the percentages of DX5+ immune subsets in bone marrow of WT and KC mice on HFCD and those of WT mice on CD could be seen (Figure S1B in Supplementary Material). The decrease in the percentages of NK cells was not due to the decline of total populations of CD45+ immune cells (Figure S1A and S1B in Supplementary Material) or total numbers of cells dissociated from different tissue compartments (Figure S2 in Supplementary Material). In assessments of spleen, pancreas, adipose, and peripheral blood, the following pattern of cytotoxicity against cancer stem cells was observed (WT/CD > WT/HFCD > KC/CD > KC/HFCD) (Figure S3A in Supplementary Material). The reverse profile was seen for the secretion of cytokines, IFN-γ, and IL-6, which were as follows (WT/CD < WT/HFCD < KC/CD < KC/HFCD) (Figures S3B,C in Supplementary Material). In contrast, bone marrow exhibited the opposite profiles of cytotoxicity and cytokine secretion, when compared to the spleen, pancreas, adipose, and peripheral blood (Figure S3D in Supplementary Material). Since IFN-γ and IL-6 secretions were increased in the cells obtained from the various tissues of KC mice on HFCD, with the exception of BM (Figures S3B–D in Supplementary Material), we aimed to determine whether other cytokines were modulated similarly or differently, using Luminex multiplex cytokine and chemokine assays. As shown in Table S1 in Supplementary Material, pancreatic cells from KC mice fed with HFCD had the highest levels of cytokine secretion. Interestingly, the secretion of cytokines was similar in WT mice on HFCD and KC on CD, and both were higher than WT on CD (Figures S3B,C and Table S1 in Supplementary Material). BM cells exhibited the inverse profiles of spleen, pancreas, adipose tissue, and peripheral blood, demonstrating less cytokine release from mice on HFCD (Figure S3D and Table S2 in Supplementary Material). PBMCs isolated from peripheral blood of mice exhibited similar results to those isolated from the pancreatic cells of four groups of mice, and KC mice fed with HFCD had the highest secretion when compared to other groups of mice (Figures S3B,C and Tables S1 and S3 in Supplementary Material). Overall, KC mice on HFCD showed the highest levels, followed by KC mice fed with CD. WT mice on HFCD had higher secretion of inflammatory cytokines when compared to WT on CD (Figures S3B,C and Table S3 in Supplementary Material). As indicated in Section “Materials and Methods” NK cell cytotoxicity was determined using LU 30/10^6^ cells, since this allowed us to summarize a large body of data within the scatter plots. The great agreement and relationship between cytotoxicity for different effector:target (E:T) ratios and LU 30/10^6^ is shown in Figures S4A,B in Supplementary Material.

### Decreased Tumor Killing and IFN-γ Secretion by Purified NK Cells From Spleen of KC Mice on HFCD

**Figure 1:**
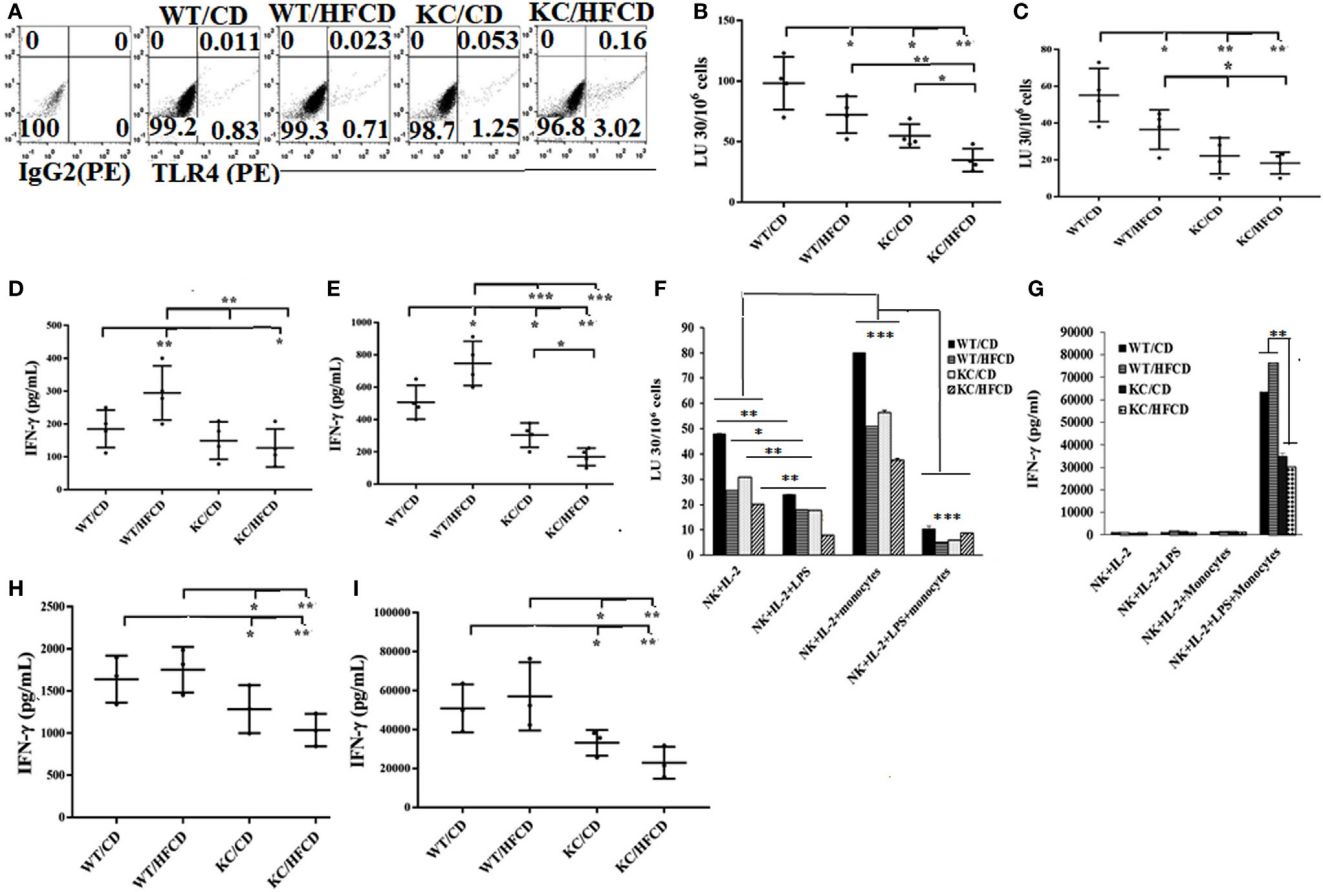
Natural killer (NK) cells from KC mice fed with control diet (CD) or high-fat calorie diet when cultured with autologous monocytes demonstrated less cytotoxicity and decreased IFN-γ secretion when compared to WT mice fed with CD. WT and KC mice were fed with a CD or high-fat calorie diet as described in Section “Materials and Methods.” Splenocytes were harvested after animals were sacrificed and single cell suspension was prepared as described in Section “Materials and Methods” and used to purify NK cells. TLR4 surface expression on the purified NK cells from four groups of mice was analyzed using antibody staining followed by flow cytometric analysis. One of three representative experiments is shown in the **(A)**. Purified NK cells from splenocytes were activated with IL-2 (10,000 U/ml) (*n* = 4) **(B,D)** or with IL-2 (10,000 U/ml) in the presence of LPS (100 ng/ml) (*n* = 4) **(C,E)** before they were used as effector cells in a standard 4-h ^51^Chromium release assay. The lytic units (LUs) 30/106 cells were determined using the inverse number of NK cells required to lyse 30% of the ST63 cells ×100 (*n* = 4) **(B,C)**, and the supernatants were harvested after culture to determine the levels of IFN-γ secretion (*n* = 4) **(D,E)**. IL-2-treated and IL-2 and LPS-treated NK cells as described in **(C,E)** were cultured with or without autologous monocytes at NK:monocytes; 1:0.5 ratio before they were used as effector cells in a standard 4-h ^51^Chromium release assay. The lytic units (LUs) 30/106 cells were determined using the inverse number of NK cells required to lyse 30% of the ST63 cells ×100. One of five representative experiments is shown in **(F)**. The supernatants were harvested to determine the levels of IFN-γ secretion. One of five representative experiments is shown in the **(G)**. IL-2-treated NK cells **(H)** and IL-2 and LPS-treated NK cells **(I)** were cultured with autologous monocytes at NK:monocytes; 1:0.5 ratio before the supernatants were harvested to determine the levels of IFN-γ secretion (*n* = 3).

The levels of NK cell cytotoxicity obtained from all the tissues examined were specific to NK cells due to the use of NK-specific target cells to determine NK cell-mediated cytotoxicity, however, the same cannot be said for IFN-γ secretion, since other immune effectors within the tissues might have contributed to IFN-γ secretion. Therefore, to establish the specific effect on the NK cells, we purified NK cells from the spleens of WT and KC mice on CD or HFCD and NK cell-mediated cytotoxicity as well as IFN-γ and IL-6 secretion were determined. Surface expression of DX5 was determined on freshly isolated NK cells (Figure S5A in Supplementary Material) and on those which were cultured (Figure S5B in Supplementary Material). No significant changes on the surface expression of DX5 could be observed in purified NK cells from four groups after culture (Figure S5B in Supplementary Material). The TLR4 surface expression was also analyzed on freshly isolated NK cells. KC mice expressed higher TLR4 expression as compared to WT mice, and KC mice on HFCD expressed higher TLR4 when compared to KC mice on CD (Figure 1A). Similar to spleen, pancreas, and adipose cells, purified NK cells from spleen activated with IL-2 (Figure 1B) or with IL-2 + LPS (Figure 1C) demonstrated the following patterns of cytotoxicity (WT/CD > WT/HFCD > KC/CD > KC/HFCD).

In contrast to secretion of IFN-γ by splenocytes, PBMCs, pancreas and adipose tissues, in which an upregulation of cytokine secretion could be seen in mice on HFCD, NK cells treated with IL-2 (Figure 1D; Tables S1, S3, S4 and S5 in Supplementary Material) or with IL-2 + LPS (Figure 1E) from KC mice fed with HFCD had the least secretion of IFN-γ, followed by KC mice fed with CD (Figures 1D,E; Table S5 in Supplementary Material). Activated NK cells from WT mice on HFCD had higher levels of secretion than WT mice on CD (Figures 1D,E). Cytotoxicity of IL-2-treated NK cells cultured with and without autologous monocytes also followed a similar trend to those shown above, except the levels of cytotoxicity by NK cells isolated from WT mice on HFCD and KC on CD were comparable; and both were more than NK cells from KC mice on HFCD (Figure 1F). Treatment of NK cells with combination of IL-2 + LPS in the absence of monocytes lowered cytotoxicity substantially when compared to NK cells treated with IL-2 alone, exhibiting the same trend in cytotoxicity as seen with IL-2-activated NK cells within the four animal groups. In the presence of monocytes an increased levels of cytotoxicity could be seen in all four groups of mice exhibiting the same trend, when compared to IL-2-activated NK cells in the absence of monocytes (Figure 1F). IL-2 + LPS-treated NK cell-mediated cytotoxicity in the presence of monocytes was substantially lower in all animal groups (Figure 1F).

When synergistic induction of IFN-γ by the purified NK cells was assessed between the four groups of mice activated with IL-2 (Figures 1G,H) or IL-2 + LPS (Figures 1G,I) with monocytes (Figure 1G), similar profiles of IFN-γ secretion, as seen with IL-2-activated NK cells in the absence of monocytes could also be observed (Figures 1G,I). Similar to PBMCs, IL-6 secretion by the NK cells was higher in mice on HFCD (please see below). T cells isolated and cultured in the same manner as NK cells, with and without monocytes, were not able to kill NK cell-specific targets (Figure S6A in Supplementary Material). In contrast to NK cells, no decrease in the induction of IFN-γ secretion by T cells activated with IL-2 + LPS and cultured with monocytes could be observed in KC mice on HFCD when compared to either WT or KC mice on CD (Figure S6B in Supplementary Material). Similar to NK cells, purified T cells from WT mice on HFCD demonstrated increased IFN-γ secretion when compared to WT mice on CD (Figure S6B in Supplementary Material).

### Osteoclasts (OCs) From KC Mice on HFCD Are Defective in Increasing Numbers and Killing of Allogeneic NK Cells From WT Mice

**Figure 2:**
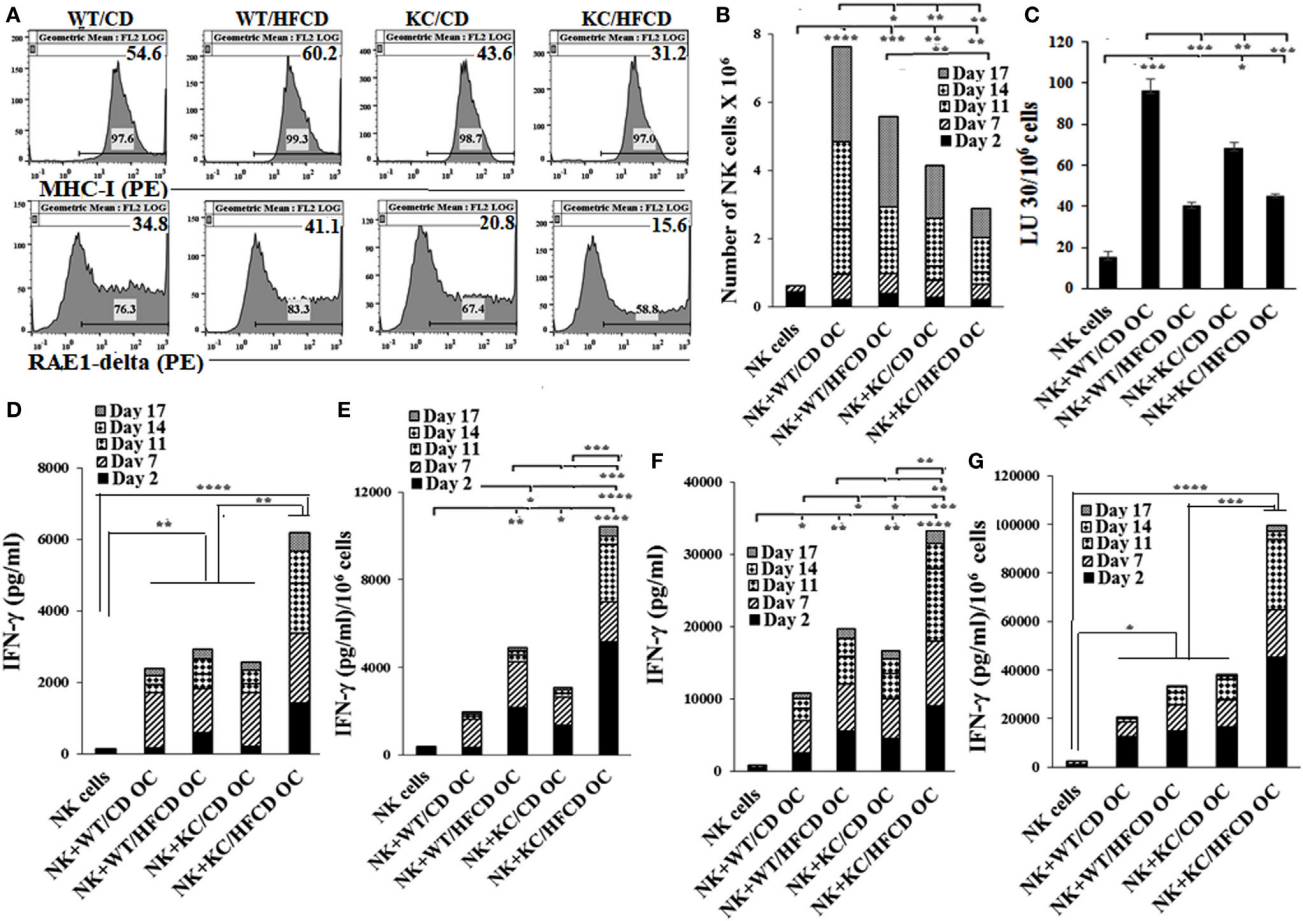
Allogeneic natural killer (NK) cells from WT mice fed with control diet (CD) expanded less and mediated less cytotoxicity, but demonstrated augmented IFN-γ secretion when cultured with osteoclasts from KC mice fed with high-fat calorie diet. WT and KC mice were fed with a CD or high-fat calorie diet as described in Section “Materials and Methods.” Bone marrow was harvested after animals were sacrificed and single cell suspension was prepared as described in Section “Materials and Methods.” Monocytes were purified from the BM cells and were used to generate OCs as described in Section “Materials and Methods.” Flow cytometric analysis was used to determine expression of RAE1-delta and MHC-class I on day 21 mature OCs **(A)**. NK cells purified from splenocytes of WT mice fed with CD were activated with IL-2 (10,000 U/ml) and LPS (100 ng/ml), and cultured in the presence/absence of OCs from different mouse groups at NK:OCs; 1:0.5 ratio, and the number of expanded NK cells were determined on the days shown in the **(B)**. NK cells expanded by the OCs as described in **(B)** were used as effector cells in a standard 4-h ^51^Chromium release assay. The LUs 30/106 cells were determined as described in Figure 1B **(C)**. NK cells were purified from WT splenocytes and cultured with OCs as described in **(B,D,E)** or treated with IL-2 in the presence of sonicated AJ2 (sAJ2) and cultured with OCs at the ratio of (0.5:1:2; OCs:NK:sAJ2) **(F,G)** and the supernatants were harvested on the days shown in the figures and secretion of IFN-γ were determined. One of three representative experiments is shown in Figure 2.

Osteoclasts were differentiated from the BM-derived monocytes for each mouse group and used for the expansion and activation of allogeneic NK cells purified from the spleen of WT mice on CD. The expression of RAE1-delta, an NKG2D ligand, and MHC-class I was much less on OCs generated from KC mice as compared to WT mice (Figure 2A). KC mice fed with HFCD had the least expression of both RAE1-delta and MHC-class I when compared to either WT mice or KC mice fed with CD (Figure 2A). As shown in Figure 2B, OCs from KC mice fed with HFCD when cultured with allogeneic NK cells from WT mice on CD in the presence of LPS had the least ability to expand NK cells when compared to OCs from WT mice on CD or HFCD or KC mice fed with CD (Figure 2B). Similar results were seen when OCs were cultured with allogeneic NK cells from WT mice on CD in the presence of sAJ2 (data not shown). NK cells from WT mice when cultured with the OCs from WT or KC mice on HFCD in the presence of LPS mediated much less cytotoxicity when compared to those fed with CD (Figure 2C). Similar results were seen in the presence of sAJ2 (data not shown). In contrast, NK cells expanded with OCs from KC mice fed with HFCD treated with LPS (Figures 2D,E) and sAJ2 (Figures 2F,G) secreted the highest levels of IFN-γ when compared to those from WT mice on CD or HFCD, or KC mice on CD (Figures 2D–G). NK cells expanded by osteoclasts from both WT mice on HFCD and KC mice on CD secreted higher levels of IFN-γ when compared to those obtained from WT mice on the CD (Figures 2D–G). Accordingly, much higher levels of inflammatory cytokines and chemokines were secreted from NK cells expanded by OCs from KC mice on HFCD, followed by those expanded by osteoclasts from KC mice on CD and WT mice on HFCD (Figures 2D–G; Tables S6 and S7 in Supplementary Material).

### Secretion of IL-6 by the Peri-Pancreatic Adipose Tissue From Obese Mice Is Synergistically Increased in the Presence of Decreased IFN-γ From NK Cells

**Figure 3:**
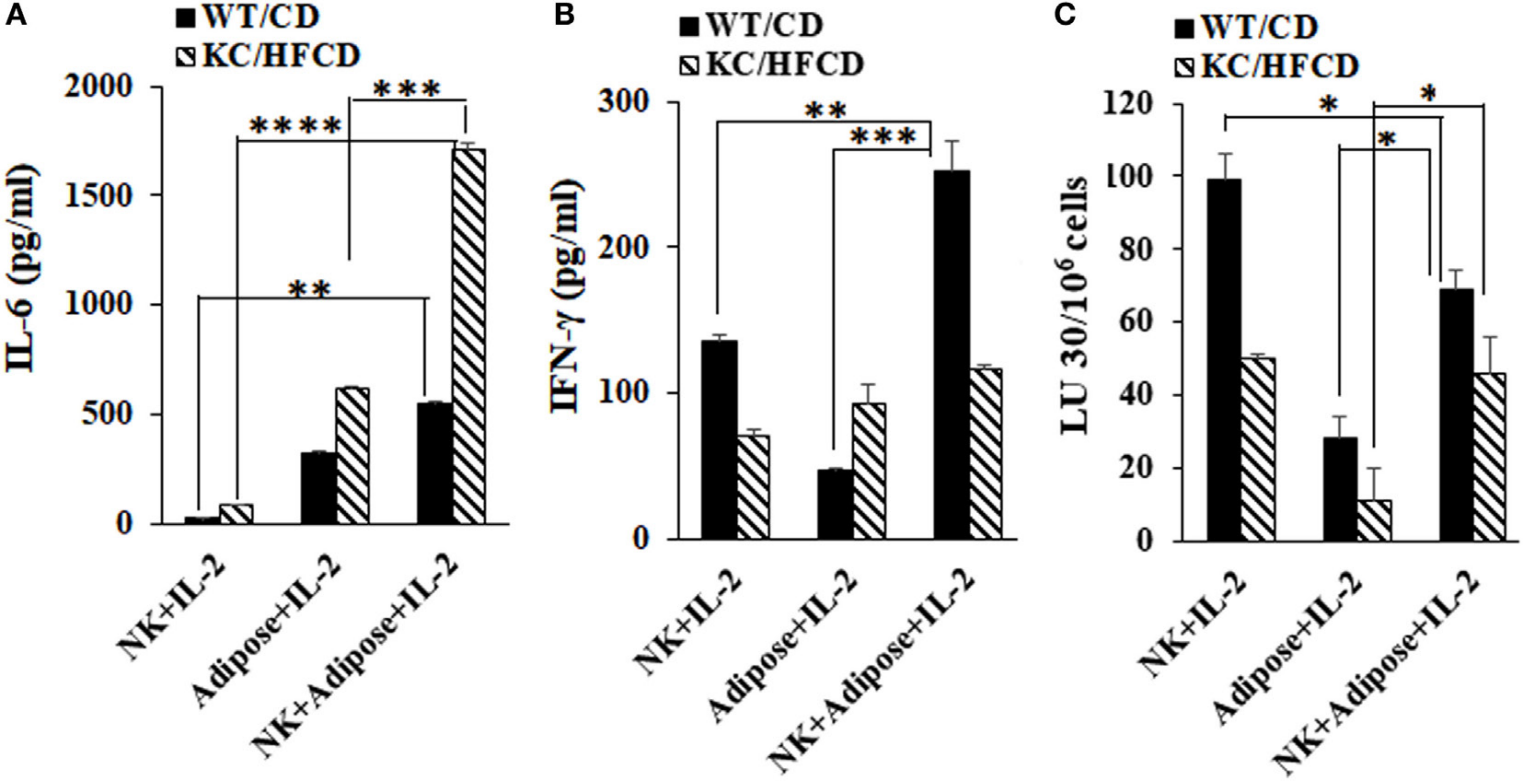
Secretion of IL-6 by the peri-pancreatic adipose tissue from obese mice is synergistically increased in the presence of natural killer (NK) cells. Purified NK cells from splenocytes and adipocytes from the peri-pancreatic fat were obtained from WT mice on CD or KC mice on HFCD as described in Section “Materials and Methods.” NK cells treated with IL-2 (10,000 U/ml) in the presence and absence of autologous adipose cells at (NK:adipose cells; 1:1) were cultured and supernatants were harvested after 7 days and the secretion of IL-6 **(A)** and IFN-γ **(B)** were determined. One of the three representative experiments is shown in the figures. Cytotoxicity of NK cells cultured as described in **(A,B)** were assessed against ST63 tumor cells using the standard 4-h ^51^Chromium release assay. The LUs 30/106 cells were determined as described in Figure 1B **(C)**. One of three representative experiments is shown in the figure.

Peri-pancreatic adipose tissue was dissociated, and the cells were cultured in the presence or absence of IL-2-activated autologous NK cells and the levels of IL-6 and IFN-γ were determined in WT mice fed with CD and KC mice fed with HFCD (Figure 3). NK cells secreted IL-6 moderately, whereas significant secretion of IL-6 could be seen by adipose-derived cells (Figure 3A). NK cells synergized with adipose-derived cells in the induction of IL-6 secretion, but the levels were substantially increased in those derived from obese mice fed with HFCD as compared to the WT mice fed with CD (Figure 3A). The inverse was seen for the secretion of IFN-γ in the cultures of NK cells with adipose tissue, in which increased amounts of IFN-γ secretion and augmented NK cell-mediated cytotoxicity, could be seen by WT mice fed with CD and less with KC mice fed with HFCD (Figures 3B,C). Thus, increased IL-6 secretion was observed in the presence of decreased NK cell-mediated cytotoxicity as well as decreased secretion of IFN-γ in KC mice fed with HFCD.

### Compared to KC Tumors, PanINs Express Lower CD44 and Are Not Susceptible to NK Cells Mediated Cytotoxicity

Single cell suspensions of PanINs and KC pancreatic tumors were obtained from KC mice and used to determine CD44 surface receptor expression and susceptibility to NK cells mediated cytotoxicity. As shown in Figure S7 in Supplementary Material, PanINs expressed relatively lower levels of CD44 expression (Figure S7A in Supplementary Material), and they were significantly more resistant to NK cells mediated cytotoxicity, when compared to either KC or ST63 tumors (Figure S7B in Supplementary Material). PBMCs were less able to lyse either PanINs or KC tumors (Figure S7B in Supplementary Material). These data indicated that PanINs are at a more differentiated stage than KC tumors.

## Discussion

The expansion and function of NK cells are known to be severely suppressed in many cancer patients (51), however, it is still unknown whether the establishment of cancer is responsible for the suppression of NK cells or lack of NK cell function at the pre-malignant, dysplastic stage is one of the driving forces for the establishment and growth of cancer or both. Studies reported in this paper are novel, since for the first time we demonstrate that NK cell loss occurs at the pre-malignant stage of cancer, and is associated with KRAS mutation in the pancreas and obesity which are likely contributing factors in the progression of cancer from dysplastic stage to overt cancer. We demonstrate that the loss of NK cell expansion and function at the pre-neoplastic stage correlates with increased levels and intensity of dysplastic lesions, increased inflammation, fibrosis, pancreatitis score, and loss of normal ductal structures (55). In addition, we also show that the combined effects of KRAS mutation in the pancreas and obesity are associated with the greater loss of NK cell cytotoxic function.

We have recently shown that NK cells specifically target and kill cancer stem cells/undifferentiated tumors, and are activated to secrete IFN-γ and TNF-α in order to drive the differentiation of tumors (63). Lack or loss of NK cell expansion and function as seen in KC mice on HFCD could likely be a contributing factor for the survival of cancer stem cells/undifferentiated tumors in addition to the failure of NK cells to promote differentiation of these tumors. We have also shown that NK-differentiated tumors proliferate or expand less, and exhibit higher expression of MHC-class I, CD54, PD-L1, and lower expression of CD44 surface receptors (69). PanINs express less CD44 and are resistant to NK cell-mediated cytotoxicity demonstrating more of a differentiated phenotype, whereas KC tumors express higher levels of CD44 and are susceptible to NK cell-mediated cytotoxicity exhibiting more of poorly differentiated phenotype (Figure S7 in Supplementary Material). Therefore, based on these profiles one may expect lower NK cell inactivation in mice at the pre-neoplastic stage than when overt cancer is established. Indeed, severe inhibition of NK cell functions is seen in pancreatic cancer patients, whereas at the pre-neoplastic stages of tumorigenesis the severity of NK cell suppression may be lower, with gradual increases in severity when the cells accumulate more mutations and become overt cancer (51). In addition, loss of NK cell-mediated cytotoxicity and severe decreases in IFN-γ secretion are found to be the reasons for pancreatic and oral tumor growth in hu-BLT mice (51, 70) and “manuscript submitted.”

When the same numbers of cells dissociated from each tissue were cultured from four groups of mice, a very consistent profile, with regards to decreased percentages of NK cells, could be observed in KC mice on HFCD. Even when gingival cells from these mice were dissociated and cultured the same profiles could be seen as the other tissues examined (55). This could be due to several factors. Since percentages of NK cells are elevated in WT mice on CD, it could be that either the tissue microenvironment is permissive for NK cell proliferation and/or that NK cells have higher cytotoxicity, and therefore they could limit and restrict survival of other immune cells and as such greater percentages of NK cells could be detected in WT mice on CD after culture. Decreased NK cytotoxicity in KC mice as compared to WT mice mirrored decreased percentages of NK cells in cells dissociated from the tissues of these mice. However, there was an inverse correlation between secretion of IFN-γ and those of NK cell-mediated cytotoxicity when total cells from the dissociated tissues were cultured (Figure S3 in Supplementary Material). Although such profiles could be due to split-anergized NK cells, in which cytotoxicity is decreased but cytokine secretion is augmented (71), it is also possible that activated NK cells may in turn activate T cells within the mixture of the cells resulting in the combined NK and T cell mediated increases in IFN-γ secretion. Indeed, due to the inactivation of NK cell function in cancer patients, we observe faster expansion of T cells which results in the decreased populations of NK cells, whereas in healthy individuals NK cells continue expanding for 30–60 days before T cell expansion takes over (51). However, when NK cells are sorted from spleen and their functions were assessed in the four groups of mice, a decrease in cytotoxicity as well as secretion of IFN-γ could be seen in autologous NK cells from KC mice. Decrease in secretion of IFN-γ by autologous NK cells could be due to the extent of defect in NK cells, since osteoclasts from KC mice on HFCD although severely restricted expansion of allogeneic NK cells, and NK cells exhibited decreased cytotoxicity, they showed increased secretion of IFN-γ. In contrast, monocytes from KC mice on HFCD decreased expansion, cytotoxicity, as well as IFN-γ secretion by autologous NK cells indicating perhaps more severe inactivation of autologous NK cells. Thus, depending on the inflammatory microenvironment and the extent of defect in NK cells and in those supporting their activation, different degrees of functional loss of NK cells may be seen. Indeed, we have previously reported four stages of NK cell maturation and activation depending on the competency of NK cells (72). At stage 2, cytotoxicity is suppressed but secretion of cytokines is induced which we have previously coined as split anergy in NK cells (72). At stage 3, both cytotoxicity and cytokine secretion are suppressed or lost (72). Therefore, based on these stages when NK cells from WT mice on CD are cultured with osteoclasts from KC mice fed with HFCD, NK cells are likely in stage 2, since they have no/lower cytotoxicity in the presence of increased cytokine secretion. However, when monocytes are cultured with autologous NK cells from KC mice on HFCD, majority of NK cells could likely be in stage 3, since both cytotoxicity and secretion of cytokines are suppressed. The latter profiles are also seen in pancreatic cancer patients in whom both NK cell-mediated cytotoxicity and secretion of cytokines are compromised.

Osteoclasts from KC mice on HFCD express much lower levels of MHC-class I inhibitory ligands and RAE1-delta activating ligands, suggesting that both inhibitory and activating ligands for signaling of NK cells are decreased. The loss of expression was much more severe on KC mice on HFCD as compared to KC mice on CD and the highest expressions were seen on the surface of WT mice on HFCD or CD. The decreased levels of MHC-class I and RAE1-delta detected on OCs correlated with the generation of PanINs in KC mice, indicating that the loss of surface receptors on OCs in combination with decreased expansion and function of NK cells may be a better indicator of PanIN induction.

In contrast to other tissues, bone marrow from KC mice demonstrated higher NK cytotoxicity in the presence of lower IFN-γ secretion (Figure S3 in Supplementary Material). At the moment, it is unclear why NK cells from bone marrow behave differently when compared to the other tissues examined, however, since NK cells develop and mature within BM it is possible that the microenvironment provides the primary activating signals which drive their initial activation, and when such activated NK cells exit to the periphery, additional signals from the microenvironment drive them to second, third, and fourth stages of NK cell maturation depending on the levels and intensities of signals they receive.

Since loss of both cytotoxicity and cytokine secretion can be seen in NK cells in KC mice on HFCD, with the highest surface receptor expression for TLR4 (Figure 1A), it may be that the defect in NK function is not at the level of availability of TLRs on NK cells, but at the levels of ligand and/or post receptor signaling. Delineating the defects at the level of post receptor signaling for TLR4 on NK cells as well as the binding and function of other key receptors on NK cells from these mice will be part of our future studies.

**Figure 4:**
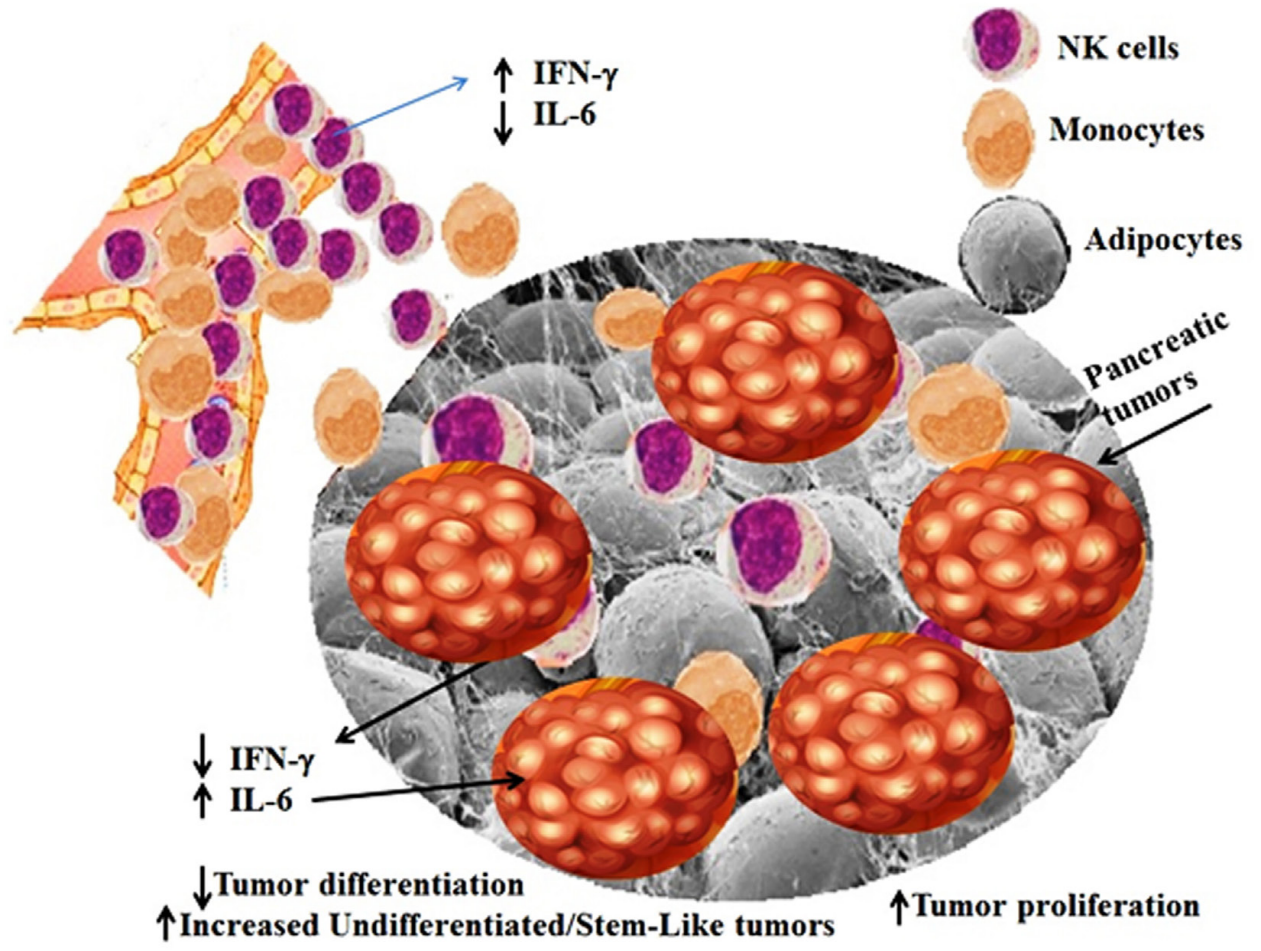
Schematic representation of hypothetical model of interaction between NK cells, adipose tissue, and pancreatic tumors. Adipose tissue within and around tumor microenvironment can contribute to the increased proliferation of pancreatic tumors through the increase in induction of IL-6 in the presence of decreased IFN-γ secretion by the NK cells. NK cells and monocytes are recruited to the peri-pancreatic and pancreatic adipose tissue from the circulation, where they are conditioned by the adipose tissue to lose secretion of IFN-γ, while increasing synergistically secretion of IL-6 by the peri-pancreatic and pancreatic adipose-derived cells. Since pancreatic tumors have a predilection to grow in the adipose tissue, these tissues are likely to convert tumor suppressive NK cells, to tumor-promoting cells, by decreasing NK-mediated cytotoxicity and IFN-γ secretion and allowing an increased secretion of IL-6 within the tumor microenvironment. Increased IL-6 secretion is instrumental in aiding in tumor growth and expansion.

Synergistic increase in the IL-6 secretion in KC/HFCD as compared to WT/CD by the peri-pancreatic adipose-derived cells with NK cells in the presence of decreased IFN-γ secretion (54% decrease in KC/HFCD as compared to WT/CD), could be one mechanism by which the adipose tissue can contribute to the increased proliferation of pancreatic tumors. Indeed, IL-6 is one of the major drivers of PDAC proliferation (73–77) and suppression of NK function (78). Moreover, addition of IL-6 to tumor/NK cultures inhibited NK-mediated IFN-γ secretion in our previous studies (42, 69, 71, 79, 80). Therefore, since tumors have a predilection to grow in the adipose tissue, these tissues are likely to convert tumor suppressive NK cells, to tumor-promoting cells. Blocking IL-6 may not only inhibit tumor growth but also it may rescue NK cells from suppression mediated by the adipose tissue or tumor tissues and offer an attractive and effective therapeutic strategy to target pancreatic tumors (Figures 3 and 4).

Our studies support the long-standing notion that both genetic and environmental factors are important in tumorigenesis, and places NK cells within the list of potential factors for the establishment and progression of pancreatic cancers, since NK cells are the main effectors capable of eliminating and differentiating cancer stem like/undifferentiated tumor.

## Ethics Statement

Animal studies were approved by the Chancellor’s Animal Research Committee of the University of California, Los Angeles in accordance with the NIH Guide for the Care and Use of Laboratory Animals.

## Author Contributions

KK performed the majority of experiments, data analysis, and preparation of the manuscript. H-HC was responsible for the breeding and feeding of the KC and WT mice and histological analysis of the tissues. PT and JC assisted KK in performing experiments and in the preparation of the manuscript. AB assisted in preparation and editing of the manuscript. GE oversaw the work with breeding and feeding of KC and WT mice and assisted in the preparation of the manuscript. AJ oversaw the overall design of the experiments, data analysis, and preparation of the manuscript.

## Conflict of Interest Statement

The authors declare that the research was conducted in the absence of any commercial or financial relationships that could be construed as a potential conflict of interest.
