# Supplementary material for: Deficiencies in Natural Killer Cell Numbers, Expansion, and Function at the Pre-Neoplastic Stage of Pancreatic Cancer by KRAS Mutation in the Pancreas of Obese Mice
Source: Front Immunol. 2018 Jun 21;9:1229. doi: 10.3389/fimmu.2018.01229 (PMC6021953; doi:10.3389/fimmu.2018.01229)

Supplementary data:

**Table legends**

**Table S1: Cytokines, chemokines, growth factors and ligands secreted by the dissociated cells from the pancreas**

WT and KC mice were fed a control diet and high-fat calorie diet as described in material and methods. Pancreas was harvested after the animals were sacrificed and single cell suspensions were obtained as described in the material and methods. Cells isolated from pancreas were cultured in the presence of IL-2 (10,000 U/mL). After culturing pancreas cells for 7 days the supernatants were harvested, and multiplex assay was used to determine cytokines, chemokines and growth factors secreted by the pancreatic cells. One of 5 representative experiments is shown here.

**Table S2: Cytokines, chemokines, growth factors and ligands secreted by bone marrow cells**

Femurs were harvested after the animals were sacrificed and single cell suspensions of bone marrow cells were obtained as described in material and methods. Bone marrow cells were cultured in the presence of IL-2 (10,000 U/mL). Supernatants were harvested after seven days of culture, and multiplex assay was used to determine cytokines, chemokines, growth factors and ligands secreted by the BM cells. One of 5 representative experiments is shown here.

**Table S3 and S4: Cytokines, chemokines, growth factors and ligands secreted by PBMCs.**

PBMCs were isolated from peripheral blood as described in the material and methods section, and were cultured in the presence of IL-2 (10,000 U/mL). Supernatants were harvested after seven days of culture, and multiplex assay was used to determine cytokines, chemokines, growth factors and ligands secreted by the PBMCs. One of 5 representative experiments is shown.

**Table S5: Cytokines, chemokines and growth factors and ligands secreted by NK cells isolated from splenocytes**

Spleens were harvested after sacrificing the animals and NK cells were purified from splenocytes using an NK negative selection kit. Purified NK cells were then cultured in the presence of IL-2 (10,000 U/mL). Supernatants were harvested after seven days of culture, and multiplex assay was used to determine cytokines, chemokines, growth factors and ligands secreted by the NK cells. One of 5 representative experiments is shown.

**Table S6 and Table S7. Cytokines, chemokines, growth factors and ligands secreted by WT NK cells when cultured with OCs obtained from the four groups of mice**

The femurs were harvested after sacrificing the animals, and bone marrow cells were obtained as described in material and methods. Monocytes were purified from bone marrow cells using negative isolation kits. Mouse osteoclasts (OCs) were generated as described in materials and methods. Purified NK cells from the spleen of WT mice were used. Purified NK cells were left untreated or pre-treated with IL-2 (10,000 U/mL) for 18 hours and then cultured alone or with mouse OCs (NK:OC, 2:1) and the cultures were either left untreated or treated with LPS (100 ng/mL). Supernatants were harvested after seven days of culture, and multiplex assay was used to determine cytokines, chemokines, growth factors and ligands secreted by the NK cells. One of 3 representative experiments is shown here.

**Figure legends**

**Figure S1: Severely decreased numbers of NK cells are associated with high-fat calorie diet and/or KRAS mutation in KC mice.**

WT and KC mice were fed a control diet or high-fat calorie diet as described in materials and methods section. The splenocytes, PBMCs, pancreas and adipose tissues were harvested after sacrifice and single cell suspensions from each tissue were prepared as described in material and methods section. The dissociated cells from each tissue sample (1X10^6^ cells/ml for total of 3 ml) were cultured with IL-2 (10,000 U/ml) for seven days and cells were stained with the respective PE- and FITC-conjugated mouse antibodies. The percentages of DX5+ expressing cells within the CD45+ immune cells were determined for each group of mice (n=3) **(A)**. The bone marrows were harvested after sacrifice and single cell suspensions from each tissue were prepared as described in material and methods section. The dissociated cells from each tissue sample (1X10^6^ cells/ml for total of 3ml) were cultured with IL-2 (10,000 U/ml) for seven days and the cells were stained with the respective PE-FITC-conjugated mouse antibodies. The percentages of DX5+ expressing cells within CD45+ immune cells were determined for each group of mice (n=3) **(B)**.

**Figure S2: No or slight differences were obtained in total cell numbers cultured from four groups of mice**

PBMCs recovered from peripheral blood (n=3), and the single cell suspensions obtained from the pancreas (n=3), adipose tissue (n=3) and bone marrow (n=3) were cultured in the presence of IL-2 (10,000 U/ml) for seven days. The total cell increase or decrease between the WT mice on CD and those of WT mice on HFCD, KC mice on CD and KC mice on HFCD were determined by dividing the number of cells obtained from each subset over those obtained from WT mice on CD **(A)**. On day seven, surface expression of CD3 was determined within the CD45+ immune cells in the adipose cells **(B)**.

**Figure S3: Decrease in cytotoxicity and augmented secretion of IFN-γ in pancreas, spleen, peripheral blood and adipose tissue, but not in bone marrow is associated with high-fat calorie diet and/or KRAS mutation in KC mice**.

Splenocytes (n=3), pancreas (n=3) and adipose (n=3) tissues as well as PBMCs (n=4) were obtained from WT and KC mice on CD, or HFCD and cultured as described in Fig. S1. Cells were then used as effectors against ^51^Cr labeled ST63 cells at various effector to target ratios in a standard 4-hour ^51^Cr release assay. The lytic units (LUs) 30/10^6^ cells were determined using the inverse number of cells required to lyse 30% of the ST63 cells X100 **(A)**. Cells were dissociated and cultured as described in Fig. S1, and supernatants were harvested and used to determine IFN-γ secretion in splenocytes (n=4), pancreas (n=5) and adipose (n=5) tissues as well as PBMCs (n=4) **(B).** Cells were dissociated and cultured as described in Fig. S1, and supernatants were harvested and used to determine IL-6 secretion in pancreas and adipose tissues as well as PBMCs. One of the three representative experiments is shown in the figure **(C)**. Bone marrows were obtained from WT and KC mice on CD or HFCD and cultured as described in Fig. S1. Cells were then used as effectors against ^51^Cr labeled ST63 cells at various effector to target ratios in a standard 4-hour ^51^Cr release assay. The lytic units (LUs) 30/10^6^ cells were determined using the inverse number of cells required to lyse 30% of the ST63 cells X100 (n=3). Supernatants were harvested and used to determine IFN-γ (n=5) secretion and IL-6 secretion. One of the three representative experiments is shown for IL-6 results **(D)**.

**Figure S4: Agreement between the levels of cytotoxicity at different Effector:Target ratios and LU30/10^6^ cells**

Splenocytes were obtained from WT and KC mice on CD and HFCD as described in Fig. S1a and were cultured in the presence of IL-2 (10,000 U/mL) for seven days. Afterwards, the cells were used as effectors against ^51^Cr labeled ST63 cells at various effector to target ratios in a standard 4-hour ^51^Cr release assay. Data presented as the mean percent cytotoxicity ± standard deviation (SD) were determined from duplicate or triplicate wells **(A)**. The lytic units (LUs) 30/10^6^ cells were determined using the inverse number of cells required to lyse 30% of the ST63 cells X100 **(B)**. One of 20 plus representative experiments is shown here.

**Figure S5: Expression of DX5 on purified NK cells**

NK cells were isolated from splenocytes of WT mice using a mouse NK cell isolation kit, as described in the material and methods section. Surface expression of DX5 was determined on freshly isolated NK cells after DX5 antibody staining followed by flow cytometric analysis (**A**). The surface expression of DX5 and CD3 were determined for the cultured NK cells treated with IL-2 (10,000 U/ml) (**B**). One of 5 representative experiments is shown here.

**Figure S6: T cells were not able to kill NK specific targets and no decrease in the induction of IFN-γ secretion by T cells could be observed in KC mice on HFCD when compared to either WT or KC mice on CD**

T cells were positively isolated from splenocytes and cultured with IL-2 (1000 U/ml) and IL-2 (1000 U/ml) in the presence of LPS (100 ng/ml) with/without autologous monocytes at a ratio of T:monocytes; 1:0.5 for seven days before they were used as effectors against St63 in a standard 4-hour ^51^Chromium release assay. The lytic units (LUs) 30/10^6^ cells were determined using the inverse number of T cells required to lyse 30% of the ST63 cells X100 **(A)** and the supernatants from day seven cultures were harvested and the levels of IFN-γ secretion were determined **(B)**. One of 3 representative experiments is shown here.

**Figure S7: Increased expression of CD44, and susceptibility of KC tumors to NK cell mediated cytotoxicity when compared to PanINs**

Surface expression of CD44 on KC, PanINs and ST63 tumors were determined using antibody staining followed by flow cytometric analysis. IgG2 isotype control antibody was used as control **(A)**. Spleen and peripheral blood were harvested from WT mice on CD, and the NK cells were purified from the splenocytes. NK cells and PBMCs were treated with IL-2 (10,000 U/mL) for 18-20 hours before they were used as effector cells in a standard 4 hour ^51^Cr release assay against KC, ST63 and PanINs. The LUs 30/10^6^ cells were determined using inverse number of NK cells or PBMCs required lysing 30% of target cells X100 **(B)**. One of 3 representative experiments is shown here.

**Table S1 (Units are pg/ml)**

**
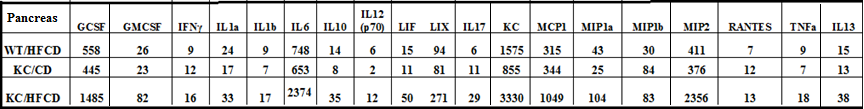
**

**Table S2 (Units are pg/ml)**


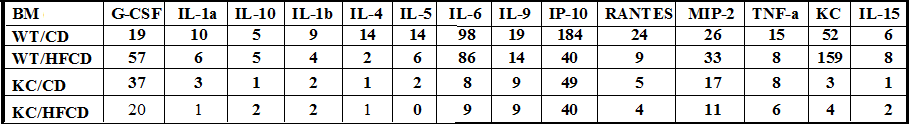


**Table S3 (Units are pg/ml)**


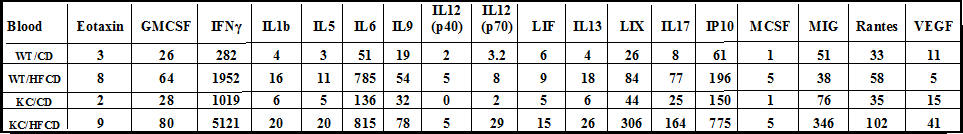


**Table S4 (Units are pg/ml)**


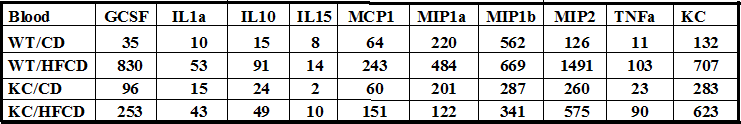


**Table S5 (Units are pg/ml)**


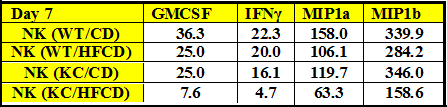


**Table S6 (Units are pg/ml)**


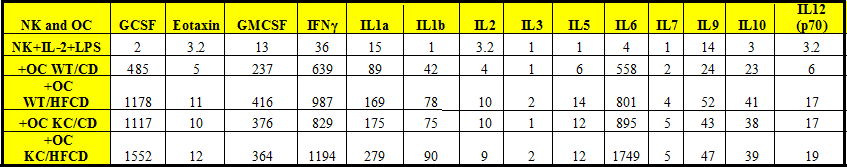


**Table S7 (Units are pg/ml)**


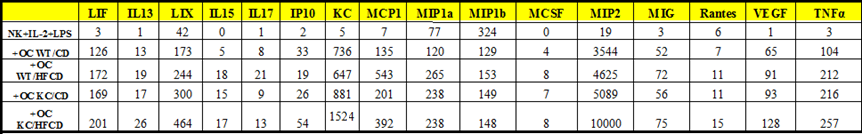


**Fig. S1**

**Fig. S1A**

**
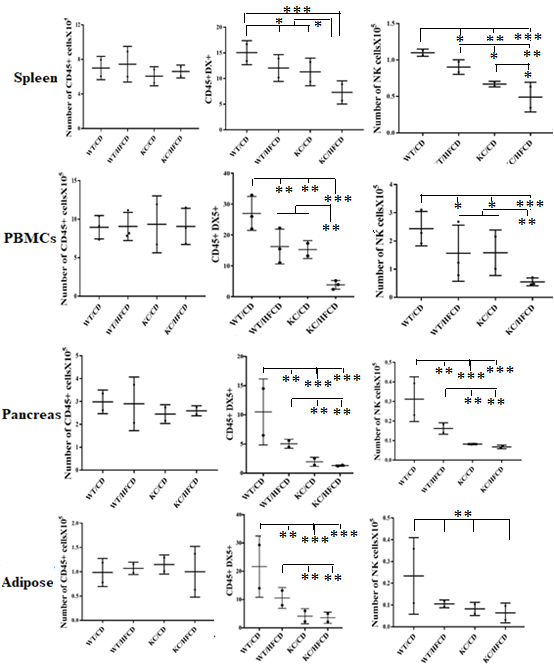
**

**Fig. S1B**

**
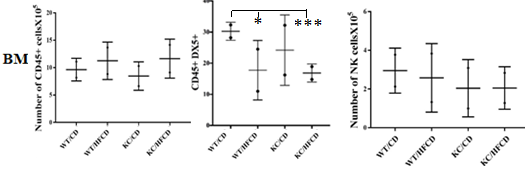
**

**Fig. S2**

**Fig. S2A**

**
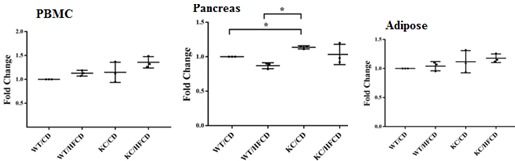
**

**Fig. S2B Fig. S2C**

**
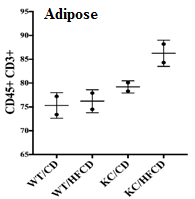
**

**
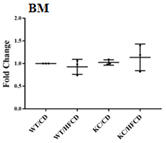
**

**Fig. S3**

**Fig. S3A**

**
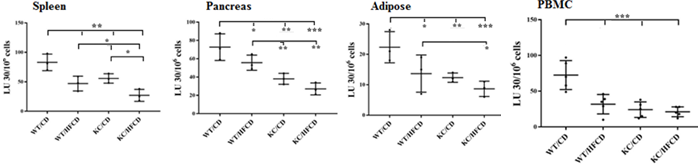
**

**Fig. S3B**

**
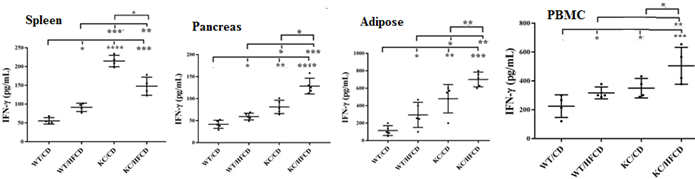
**

**Fig. S3C**

**
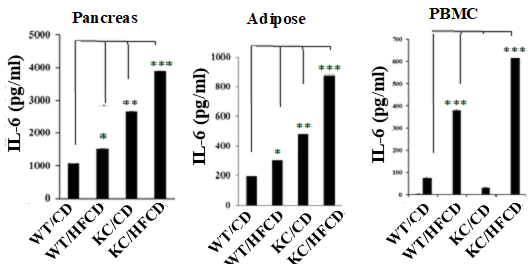
**

**Fig. S3D**

**
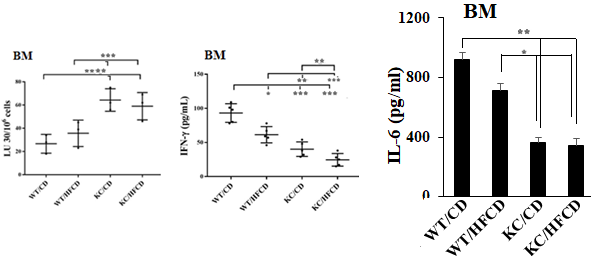
**

**Fig. S4**

**Fig. S4A**

**Fig. S4B**

**Fig. S5**

**Fig. S5A**

**
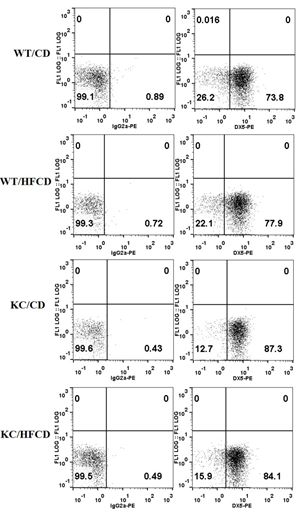
**

**Fig. S5B**

**
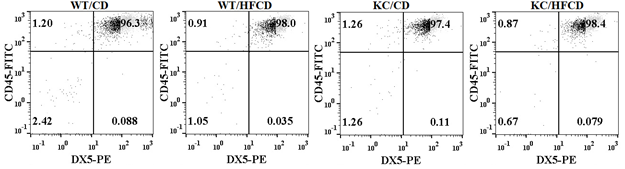
**

**Fig. S6**

**Fig. S6A**

**
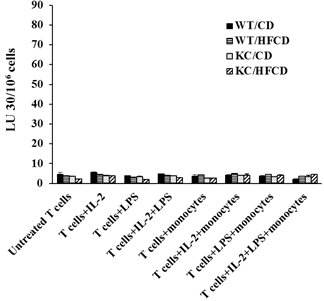
**

**Fig. S6B**

**
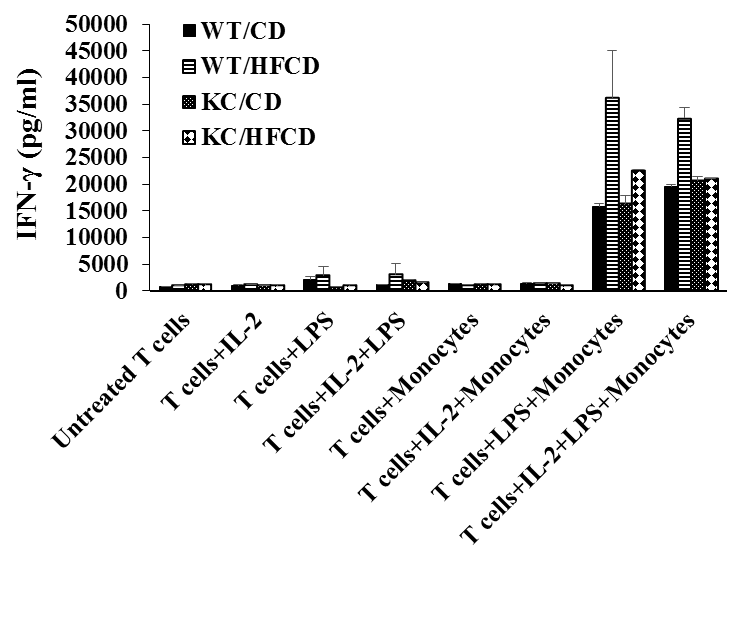
**

**Fig. S7**

**Fig. S7A Fig. S7B**


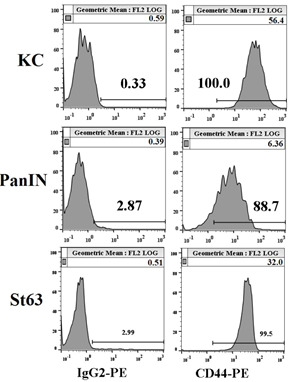

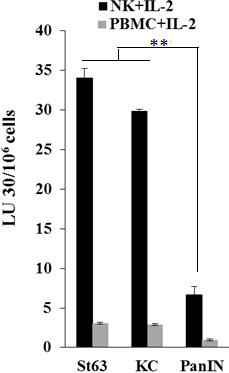

Supplement: Supplementary file 1 [file Data_Sheet_1.docx]
